# The dynamic wound microbiome

**DOI:** 10.1186/s12916-020-01820-6

**Published:** 2020-11-24

**Authors:** Chunan Liu, Alise J. Ponsero, David G. Armstrong, Benjamin A. Lipsky, Bonnie L. Hurwitz

**Affiliations:** 1 Department of Biosystems Engineering, University of Arizona, Tucson, AZ USA; 2 BIO5 Institute, University of Arizona, Tucson, AZ USA; 3 Department of Surgery, Southwestern Academic Limb Salvage Alliance (SALSA), Keck School of Medicine of University of Southern California, Los Angeles, USA; 4 Department of Medicine, University of Washington, Seattle, WA USA; 5 Division of Medical Sciences, Green Templeton College, University of Oxford, Oxford, UK

**Keywords:** Diabetic foot ulcer, Wound microbiome, Metagenomics, Next-generation sequencing

## Abstract

**Background:**

Diabetic foot ulcers (DFUs) account for the majority of all limb amputations and hospitalizations due to diabetes complications. With 30 million cases of diabetes in the USA and 500,000 new diagnoses each year, DFUs are a growing health problem. Diabetes patients with limb amputations have high postoperative mortality, a high rate of secondary amputation, prolonged inpatient hospital stays, and a high incidence of re-hospitalization. DFU-associated amputations constitute a significant burden on healthcare resources that cost more than 10 billion dollars per year. Currently, there is no way to identify wounds that will heal versus those that will become severely infected and require amputation.

**Main body:**

Accurate identification of causative pathogens in diabetic foot ulcers is a critical component of effective treatment. Compared to traditional culture-based methods, advanced sequencing technologies provide more comprehensive and unbiased profiling on wound microbiome with a higher taxonomic resolution, as well as functional annotation such as virulence and antibiotic resistance. In this review, we summarize the latest developments in defining the microbiology of diabetic foot ulcers that have been unveiled by sequencing technologies and discuss both the future promises and current limitations of these approaches. In particular, we highlight the temporal patterns and system dynamics in the diabetic foot microbiome monitored and measured during wound progression and medical intervention, and explore the feasibility of molecular diagnostics in clinics.

**Conclusion:**

Molecular tests conducted during weekly office visits to clean and examine DFUs would allow clinicians to offer personalized treatment and antibiotic therapy. Personalized wound management could reduce healthcare costs, improve quality of life for patients, and recoup lost productivity that is important not only to the patient, but also to healthcare payers and providers. These efforts could also improve antibiotic stewardship and control the rise of “superbugs” vital to global health.

## Background

Chronic wounds are a common complication of diabetes mellitus that can severely affect a patient’s quality of life and may lead to lower limb amputation [1–5]. The 5-year mortality for diabetic foot ulcers (DFUs), and minor and major amputations was recently reported to be 30.5, 46.2, and 56.6% [6]. In particular, foot ulcers are prevalent in patients with long-standing diabetes, mostly related to peripheral neuropathy and ischemia from peripheral vascular disease [7]. These DFUs are always colonized by microorganisms and often become infected by pathogenic microbes, resulting in a diabetic foot infection (DFI). It is estimated that 44–68% of patients admitted to the hospital with a DFI will develop diabetic foot osteomyelitis (DFO) which often requires surgical treatments and a long antibiotic therapy [8–10]. Further, biofilm-producing microbes delay healing and underlie up to 90% of chronic wounds [11]. Detecting when a DFU is infected is often difficult due to a lack of local or systemic signs or symptoms, primarily related to the perturbed neurological, vascular, and local inflammatory responses in these patients [12–15]. The standard approach for identifying microorganisms colonizing a wound is to obtain a specimen (preferably of tissue or bone) for aerobic and anaerobic culture. However, achieving results with this time-honored approach usually takes several days (up until 14 days) and is biased toward a subset of microbes that grow well in the laboratory setting. Furthermore, the likelihood of false-negative cultures increases when patients are treated with antimicrobials or clinicians employ inadequate sampling methods, especially superficial swabs of open wounds. False positives can result due to an inability in distinguishing pathogens from healthy skin flora or contaminants. Culture-based approaches have also been found to fail to identify most fungi and frequently do not accurately represent the complete bacterial communities present in the wound [16]. Hence, treatment based on the results of standard culture may fail to cover one or more important pathogens in a DFI [17].

These deficiencies of standard culture have led researchers to seek more technologically advanced methods to identify pathogens from infected wounds. Recently, newer sequencing methods have better defined the surprising microbial diversity found in many areas of the human body (“human microbiome”) and the dynamic status of these microorganisms [18–20]. These technologies have provided extensive descriptions of the microbiomes of the gastrointestinal tract and, more recently, the skin [21]. A better understanding of the skin microbiome, which generally holds the organisms responsible for DFIs, should advance our diagnostic and therapeutic approaches to DFIs. Moreover, the characterization of the microbiome associated with osteomyelitis of diabetic foot is also critical as the bone can harbor a bacterial community distinct from the skin. The microbiological spectrum of foot osteomyelitis in diabetic patients is similar to that of the contiguous soft-tissue infection but fewer numbers of isolates are usually found in bone [8]. Sequencing technologies have detected significantly more anaerobes and Gram-positive bacilli in bone samples compared to conventional techniques, which may contribute to the poor success rate of medical treatment of DFO [9]. Thus, we herein review the latest developments in defining DFU microbiology that has been unveiled by sequencing technologies and discuss both the future promises and current limitations of these approaches. Moreover, we focus on how these technologies can be applied to monitoring and measuring wound progression.

## Molecular techniques and limitations

Thanks to increasingly affordable sequencing platforms and the development of rapid and efficient bioinformatics tools, there have been an increasing number of culture-independent (molecular) studies for characterizing various human microbiomes (summarized in Table 1). Compared to culture-based techniques, DNA sequencing of the small subunit ribosomal RNA (SSU rRNA) gene and metagenomic next-generation sequencing (mNGS) provide a comprehensive and precise description of the microbial community. These studies have demonstrated that the vast majority of microorganisms in these microbiomes are not detected by standard culture methods, while molecular techniques allow the characterization of microbial populations in their environment using the genetic content of the entire community [22]. Importantly, culturomics approaches have been developed to address the limitations of classical culture methods to increase throughput and allow for the identification of unknown bacteria through 16S rRNA sequencing as reviewed in Lagier et al. [23]. Culturomics has been successfully applied to several human-associated microbial communities, and Jneid et al. [24] have confirmed its complementary role in relation to molecular methods in the exploration of complex microbiota in DFIs.

**Table 1:** Advantages and limitations of approaches for clinical diagnosis of microbes

Technique	Definition	Pros	Cons	Used in clinics
Conventional culture	Growth in culture to isolate a pure sample followed by phenotypic analysis.	Identification of features such as the ability to grow on specific culture mediums, antibiotic resistance, and biochemical attributes such as the ability to alter particular substrates.	Possible only for a small fraction of organisms, and highly variable growth rate may lead to biases in detection. Additionally, the method is slow and takes 48–72 h to generate results.	Yes
Microscopy	Microscopy-based approach that utilizes a short culture period followed by fluorescence-based tagging of microbes with antibodies and DNA probes. The sample then flows across the microscope field with automated computer-based identification of both microbial shape and automated fluorescence detection.	A large number of samples can be processed rapidly.	Biases in culture approaches to enrich the microbes, and in detection. Difficult to adapt to non-bacterial pathogens.	Yes
Mass spectrometry	Matrix-assisted laser desorption ionization-time of flight (MALDI-TOF) mass spectrometry for the identification of bacteria. A pure culture is ionized and the mass spectra of the resulting protein fragments compared to a reference database to match patterns of known organisms. Recent advances to the method allow cruder preparations to be analyzed and even negate the need for culture.	Rapid and low-cost once the initial equipment has been installed.	Difficulty in detecting drug resistance and virulence, and in detecting viruses, fungi, and parasites. Additionally, complex mixtures of organisms can present an unsolvable mass spectrum making its utility best suited to scenarios in which only a single organism is expected.	Yes
Polymerase chain reaction	Sequencing of specific unique sequences in the genomes or transcriptomes of organisms, leading to the creation of an amplicon used to detect the presence of that organism. Additionally, primers can be designed to indicate the presence of drug resistance and virulence gene sequences.	Rapid and low-cost.	Limited range of organisms that can be identified. Inability to identify organisms that are not present in the panel design, and the assumption that target sequences is unique to a particular organism.	Yes
Microarray	Multiple DNA probes are fixed to a solid surface and hybridized to sample DNA fragments that are modified (typically with fluorescent tags or a means to generate fluorescence following hybridization) that allow detection of probes with hybridized sample DNA.	Ability to detect and identify a broad range of organisms and their drug resistance and virulence sequences in a single assay. Can be used to analyze mixtures of organisms.	Difficulties in designing appropriate probes, difficulties in distinguishing organisms at the strain level. Issues of specificity and time required to perform the assay.	No
DGGE/TGGE on the SSU rRNA gene	Molecular fingerprinting targeting the SSU rRNA gene.	Rapid visualization of prokaryotes community changes.	Do not allow the identification of the specific prokaryotic species involved.	No
16S rRNA sequencing	Amplicon sequencing on the SSU rRNA gene.	Allow the identification and characterization of microbial diversity.	Do not allow strain-level description of the community, do not take into account the viral and eukaryotic fraction of the population.	No
Metagenomic next-generation sequencing (mNGS)	DNA is sheared randomly into small segments and sequenced.	Taxonomic identification of bacteria to the species or strain level, detection of virulence factors and antibiotic-resistance genes.	High amounts of host DNA contamination, high cost of sequencing.	No

### Amplicon sequencing

Most studies of the microbiology of DFU are based on the amplification of the small subunit ribosomal RNA (SSU rRNA; 16S rRNA for bacteria and archaea, 18S rRNA for eukarya). The SSU rRNA gene is highly conserved; however, variations in hypervariable regions can be used to distinguish microbial species. Molecular fingerprinting methods targeting the SSU rRNA gene, such as denatured gradient gel electrophoresis (DGGE) or a temperature gradient gel electrophoresis (TGGE), are frequently used to rapidly identify microbes and monitor differences in composition over space or time [25]. These methods separate DNA fragments of equal length based on their sequence melting point. Therefore, compositional diversity can be rapidly visualized using these methods where each band on a gel represents a specific prokaryotic taxon, although in certain cases, two different species can migrate at the same length, leading to possible misidentifications. Although DGGE and TGGE can be used for better characterization of complex microbial flora than culture-based methods, the identification of the specific prokaryotic species present in the sample can be difficult.

Amplicon sequencing allows for the identification and characterization of microbial diversity and to identify microbial community members using amplification, sequencing, and analysis of the SSU rRNA gene. Older studies relied on Sanger sequencing methods to obtain full-length SSU rRNA gene sequences, but because of the cost and sequencing capacity, only for a small subset of the organisms in a sample. Next-generation sequencing (NGS) techniques have vastly improved sequencing depth, but have shorter (~ 500 bp) sequences and therefore typically use one or more hypervariable regions as a proxy for the full-length gene.

Amplicon-based studies on DFU samples reveal a much more complex bacterial community associated with DFU than those identified by culture [26, 27] and provide some insight into patient outcomes for these wounds [28, 29]. However, these approaches are limited by amplification biases, as no universal SSU rRNA primers exist, and the primer choice affects the amplification efficiency of different microbial phyla. Furthermore, the quality of DNA extraction varies according to the microbial taxa [30]. Importantly, these methods are restricted to prokaryotes and fungi and do not account for bacteriophages and eukaryotic viruses, which have been shown to modulate virulence and biofilm formation of *Staphylococcus aureus* in DFU [31]. While 16S rRNA amplicon studies allow for the identification of microbes down to the genus level, these approaches cannot be used for species- or strain-specific characterization. While bioinformatics tools now exist to predict functional content from 16S sequences [32], these methods have been shown to be biased toward known microbial groups and pathways [30]. Additionally, these predictive analyses cannot infer antibiotic resistance and virulence genes that are of interest in clinical samples.

### Metagenomic next-generation sequencing

**Fig. 1:**
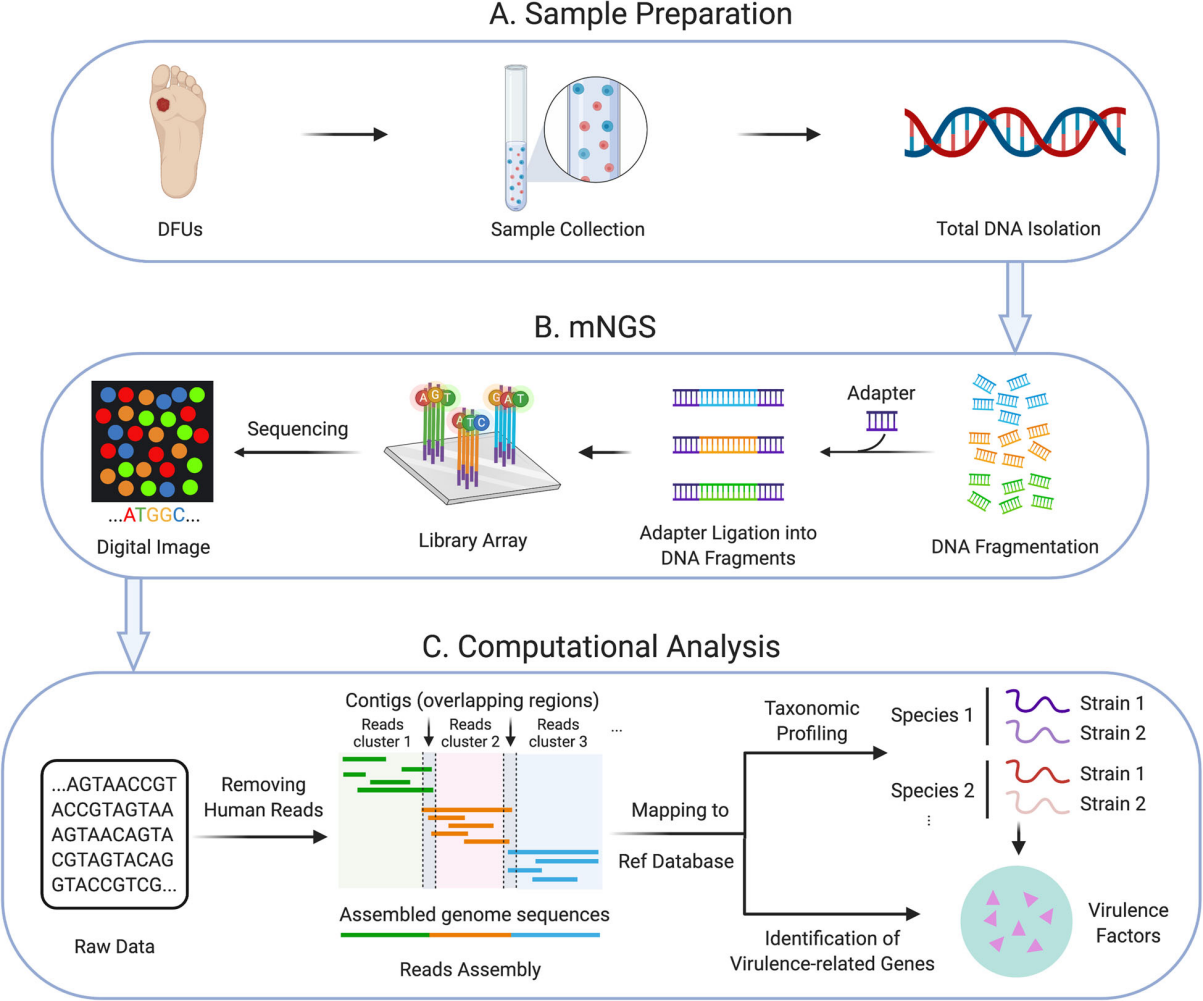
mNGS workflow. mNGS analysis mainly involves three steps: (**a**) isolation of the DNA from clinical samples, (**b**) library generation and sequencing, and (**c**) computational analysis of the sequence reads to identify the organisms and their relative abundances in a given sample, and the presence of virulence-related genes

Despite a growing number of studies using sequencing to investigate microbial communities in wounds, few use metagenomic next-generation sequencing (mNGS) approaches (mNGS workflow is shown in Fig. 1). Unlike amplicon-based sequencing methods, mNGS indiscriminately sequences all genes for all organisms in a given microbial population at their relative abundance in the sample, including viruses and other mobile elements [33]. When compared with 16S-based metagenomics, it offers a finer taxonomic identification for bacteria to the species or strain level. In their 2019 study, Kalan et al. compared 16S rRNA amplicon sequencing and mNGS results for 195 samples from 46 DFU patients [34]. Overall, each approach identified the same major bacterial genera and was significantly correlated based on taxonomic diversity in the samples. However, mNGS offered additional insights into the strain-level diversity of *S. aureus*, bacteria that have been shown to drive outcomes in patients with a DFU [34]. Moreover, mNGS allows an estimation of metabolic pathways present in the microbial population. In chronic wounds, identifying virulence factors and antibiotic-resistance markers in the microbial community can be particularly helpful in selecting antimicrobial therapy. Using mNGS, Kalan and collaborators showed that an increase in genes related to biofilm formation in the microbial population was associated with non-healing DFU [34].

In conclusion, mNGS avoids amplification bias, captures both cellular and viral components of the community, and allows for species/strain-level analysis. These datasets are, however, more expensive to generate and require greater computational knowledge and resources to store, process, and analyze. Moreover, human-associated microbiomes often contain a large amount of human DNA contamination. This is especially problematic when sampling wound tissues and bone specimens. The vast majority of reads are human in unenriched samples [35], due to human contamination from lysed human cells and free cellular DNA. Thus, samples either need to be enriched, leading to low quantities of DNA and the potential to sequence contaminants or deeply sequenced, leading to extensive costs that greatly exceed the cost for culture.

Finally, although both amplicon-based and mNGS approaches allow the identification of microorganisms in an environmental sample, unlike cultures, they do not distinguish living from dead or dormant microorganisms. For this reason, and because many chronic wound samples reveal multiple isolates, it is particularly challenging to identify which microbes are actively contributing to infection in this setting.

### Different approaches may reveal different microbial populations

Culture-based approaches for the identification and quantitation of microbes in wounds are limited by the fact that various factors impair their ability to identify the causative microorganisms that are both viable and actively infecting (rather than colonizing or contaminating) the wound. A first step in obtaining accurate results is to obtain high-quality samples to submit for culture. This begins with proper cleaning and debriding the wound (mainly performed by a specialized surgery in the operating room), then having specimens collected by an appropriately trained clinician supplied with proper equipment, taking tissue curetting or biopsies (not swabs), and ensuring the time and care to avoid procedures that might contaminate the specimen. To ensure the microorganisms in the specimen remain viable uncontaminated and viable, they must be carefully handled and quickly delivered to the microbiology lab for culturing using adapted transport methods and media. In the laboratory, specimens must be inoculated on the proper media, under appropriate conditions, and for the required duration to allow growth (especially if seeking anaerobic prokaryotes). Oftentimes, culture results identify and report organisms that easily grow in the laboratory environment, but that underestimates the diversity of the skin microbiota in samples from both healthy hosts and those with a chronic wound [16, 36]. Obligately, anaerobic prokaryotes are often present in DFUs, but they are often fastidious and are only detectable impeccable culture methods or molecular sequencing approaches [19]. Thus, cultures may have a high false-negative rate and lack full representation of the complete microbial population in wounds.

With microbiome surveys becoming more prevalent in studies of patients with chronic wounds or DFUs, it is important to compare the techniques the authors used and assess their limitations. In a 2016 study, Meisel et al. compared the effect of sequencing different 16S hypervariable regions for studying skin microbiota samples. Their results suggest that the V1–V3 region provides a more accurate taxonomic assessment than the V4 region [37]. Moreover, the authors compared the genetic predictions based on 16S rRNA sequencing and pathways annotations from mNGS datasets. The two approaches revealed similar results, suggesting that in some instances genetic prediction can provide a reasonable estimate of functional enrichment when mNGS cannot be performed.

## The dynamic DFU microbiome

### The diabetic skin microbiome

The skin microbiome is not only a complex (containing multiple species) but a highly dynamic microbial community that maintains an interdependent relationship with the human host. In a healthy state, skin commensal microbes co-exist with their human hosts and help prevent potential pathogens from colonizing the skin. In response to skin damage, the host immune system triggers an inflammatory cascade to help avert pathogen invasion and initiate the healing process. Some bacteria residing on the skin can exert antimicrobial activities against pathogens [38] or decrease the pathogen virulence [39] to protect the host. Importantly, commensal microbes can often be distinguished from pathogens based on pathogen-associated molecular patterns (PAMPs) or molecules that are associated with infectious agents in the affected tissue [40, 41]. That said, organisms that are often considered non-pathogens can cause infection in hosts with impaired immunity or who have recently received antimicrobial treatment.

When immune responses are impaired, as is often the case of persons with diabetes, they may fail to prevent colonization by pathogenic bacteria in the wounded tissue. In chronically infected wounds, many bacteria form a biofilm, in which they irreversibly attach to and grow on a surface, produce extracellular polymers that facilitate matrix formation, and alter their phenotype with respect to growth rate and gene transcription.

Of note, studies have demonstrated clinically relevant differences in the foot’s skin microbiome between non-diabetic and diabetic individuals, which likely contribute to the higher rates of skin-associated infectious diseases in diabetic patients [42]. A recent longitudinal study [43] followed patients with uninfected DFUs over a period of 10 weeks, comparing the microbial population of the DFU to the skin on the contralateral site on the same patient, as well as a control group of healthy subjects without diabetes. They found that the microbiome of the wound was different from the unaffected skin in the diabetic patients (contralateral site to the wound), and notably, the microbiome of the unaffected skin was significantly less diverse in these diabetic persons than that in non-diabetic persons. They also identified 69 operational taxonomic units (OTUs) specific to the diabetic skin microbiome that may provide further insight into variations in healing.

### DFU microbial composition and patient outcomes

Predictive analytics (using data, statistical algorithms, and machine learning techniques to identify the likelihood of future outcomes based on historical data) can help to guide treatment by identifying patterns in microbial species composition linked to patient outcomes. Several recent studies have begun to explore differentiating the microbial composition of healing and chronic wounds. In one, infected DFUs with a long duration (≥ 6 weeks) were found to have a more diverse microbiome than rapidly healing ones [44]. These severely infected DFUs had altered microbial community structures compared to mild or moderate infections, showing a stratification between wounds and their severity. Moreover, results from MacDonald et al. [45] suggested that healing DFUs had a higher abundance of *Actinomycetales* and *Staphylococcaceae*, while non-healing DFUs showed higher abundances of *Bacteroidales* and *Streptococcaceae*. Facultative anaerobes, especially of the genus *Enterobacter*, were found to be significantly associated with lack of healing, and thus a negative prognostic factor derived from knowing the chronic wound microbiome [46]. However, despite these trends, no statistically significant correlation was found between wound status (healed versus unhealed) and the abundance of any particular taxa at the species or genus level in several other studies [29, 43, 47].

Bacterial strain-level variations can also have important functional differences that influence their interactions with their host. Staphylococci isolated from DFUs have been found to be genetically diverse [48], among which *S. aureus* is a major colonizer that produces abundant biofilm and thereby inhibits wound healing resulting in wound infections [49, 50]. Kalan and colleagues found that for DFU, using mNGS showed that variations in strains of *S. aureus* were correlated with ulcer healing time [34]. Importantly, strain-level variation of *S. aureus* genes was also predictive of poor outcomes in a type 2 diabetes mouse model [34].

The occurrence of pathogenic and multidrug-resistant strains like methicillin-resistant *S. aureus* (MRSA) strains negatively influences treatment outcomes and leads to chronicity of ulcers [49, 51, 52]. Shettigar and Murali [52] recently grouped strains of *S. aureus* into clonal lineages, along with their associated virulence markers involved in skin and wound infection to help in distinguishing between colonizing and pathogenic strains. Using mNGS, determining the presence of genes encoding antibiotic resistance, and virulence factors can help in better understanding and predicting patient outcomes. Kalan et al. [34] showed that antibiotic-resistance genes are abundant in microorganisms comprising the DFU microbiome. In particular, the authors isolated a complete genome for *S. aureus* that carried multiple antibiotic-resistance genes and found it was most abundant in samples from patients with poor outcomes. Slow-healing and chronic wounds were also enriched with organisms in biofilm-related pathways, including the *agrABCD* operon that encodes genes important for biofilm development and virulence [34]. Likewise, Sloan et al. [47] identified genes for tetracycline and macrolide resistance in strains of *Streptococcus anginosus* and *Prevotella intermedia* from DFU samples. However, neither tetracyclines nor macrolides are currently commonly used by clinicians for treating diabetic foot infections, except in selected scenarios, e.g., for patients who are penicillin-allergic or for infections with pathogens that are resistant to other commonly used antibiotic agents.

Due to the polymicrobial nature of DFU, understanding the complex interactions between pathogens and the commensal flora, rather than the simple presence or absence of specific bacteria, is often more informative with respect to the evolution of the wound [53]. For example, the pathogenic effects of anaerobes can be increased by the presence of aerobes as they consume oxygen inducing tissue hypoxia, and facilitating the growth of anaerobes [54, 55]. Such symbiotic relationships are defined as the cooperative interaction of two or more species that may result in an increase in virulence leading to delayed healing [56]. *Pseudomonas aeruginosa* and *S. aureus*, the two most common causes of chronic wound infections, are frequently found together, and combined *P. aeruginosa* and *S. aureus* infections are more virulent than single infections [57–60]. Additionally, a wound model has shown that when grown in culture together, *P. aeruginosa* and *S. aureus* display an enhanced antibiotic tolerance [60]. *Bacteroides fragilis* has been reported in several studies as the predominant anaerobic bacteria isolated in DFIs [61–63], and is also important in community dynamics and biological interactions. Mastropaolo et al. [64] demonstrated the effect of polymicrobial infection involving *Escherichia coli*, *Bacteroides fragilis*, and *Clostridium perfringens* in a mouse model of type 2 diabetes.

Aside from synergistic interactions between pathogens, competitive interaction between non-pathogenic commensal bacteria and pathogens has been observed in wounds. Indeed, the presence of the commensal *Helcococcus kunzii* in wounds significantly reduced the virulence of the *S. aureus* without directly modifying the host defense response [39]. In addition, prophage inserted into the *S. aureus* host genome in a DFU also appears to attenuate bacterial virulence [31]. All in all, there is growing evidence that polymicrobial interactions may synergize the pathogenic potential or decrease the virulence of other microorganisms and have a major impact on the severity and evolution of wound infection. Therefore, it is of great importance to move beyond the presence/absence survey of bacteria in DFUs and exploring potential microbial interactions in wounds.

## Effect of medical intervention on the DFU microbiome

Effective therapeutic interventions are critical to the management of a DFU to prevent them from becoming infected or chronic, which can lead to many adverse consequences, including lower extremity amputation. Required treatments generally include adequate glycemic control as well as the debridement of compromised or necrotic tissues, offloading pressure, covering wounds with appropriate dressings, and for clinically infected wounds, administering appropriate antimicrobial therapy [65]. It is also useful to monitoring the evolution of the DFU microbiome in response to treatment to help ensure that (1) new pathogens have not emerged that are not covered by the current antibiotic treatment; (2) the antimicrobial therapy is the narrowest spectrum needed, in concordance with the principles of antibiotic stewardship [66]; and (3) the target organisms are being eradicated with the current treatment.

Of course, antibiotic treatment itself may drive major changes in wound microbiota composition [16, 45, 67, 68]. Sloan et al. [47] found a reduction in microbial diversity in DFU wounds after affected patients had been treated with doxycycline, ciprofloxacin, and metronidazole. This reduction in diversity can potentially lead to the unchecked growth of pathogens in the wound. For example, empirical treatment with doxycycline in a DFU was followed later by an expansion of streptococci, while in another wound a subsequent expansion of *Enterobacteriaceae* was observed following treatment with co-amoxiclav to an episode of clinical infection caused by *Anaerococcus* and *Peptonophilus* [47]. Other studies report contradictory evidence, where DFUs in patients with exposure to systemic antibiotics who demonstrated delayed healing remained stagnant and did not show any significant changes in terms of microbial community diversity or composition [29, 34], compared to those that healed. Loesche and colleagues propose that wounds should optimally be dynamic during the healing process, where the microbiome shifts from infected to normal skin flora [29].

Wound debridement and wound dressings have also been shown to significantly modify the wound microbiome in DFU patients, with evidence that this led to favorable outcomes [34], even for long-term chronic wounds [43]. However, Verbanic et al. [46] found no difference in the wound microbiome between pre-debridement and 1–2 min post-debridement specimens, suggesting that debridement did not alter the wound microbiome directly in the short term. Notably, in this study, the extent and depth of debridement, as well as the type of instrument used (curette, scalpel, scissors, or tissue nipper), were not standardized and were determined by the treating physician. Additionally, abrupt changes in DFU community composition have also been reported without any obvious causes, such as antibiotic exposure or the development of clinical infections [47]. Other treatments like probiotic bacteria and phage therapy are potential alternatives that may have effects on the DFU microbiome, but have not yet been well-defined [69–71].

Finally, the effect of antibiotics may be limited, especially under in light of the increasing problem of antimicrobial drug resistance. When antibiotic-resistant bacteria emerge, they are typically difficult to eradicate, contributing to the low efficiency of antibiotic therapy in DFU management [15, 41]. Additionally, many clinics rely on a traditional culture that underestimates wound flora and may lead to inappropriate antibiotics prescribed in up to 45% of cases [72, 73]. The excessive (too broad-spectrum or of unnecessarily long duration) or inappropriate (treating clinically uninfected wounds) use of antibiotics not only results in ineffective treatment but also aggravates the worldwide crisis of antibiotic resistance. mNGS holds great promise as a method to accurately and quickly detect and quantify genes related to antibiotic resistance, thus assisting clinicians to better select the most appropriate antibiotic regimen. However, long-term adoption and utility of these methods in the clinic may be limited, given the relatively long time to get a result and difficulties in convincing both clinicians and patients to change to a more targeted drug once a course of broad-spectrum antibiotics has been started.

## Moving to the clinic

Despite recent strides in research in the microbiology of DFU, using molecular approaches (16S rRNA and mNGS) to assess the composition of the microbial community, developing a clinical diagnostic algorithm and using these approaches will require first demonstrating both their clinical validity and utility. Specifically, clinical trials must not only validate molecular results compared to culture but also assess the cost-effectiveness of the resulting treatment approaches. In particular, are the added costs for molecular methods justified by providing faster and more comprehensive results that actually improve outcomes, either for the patient or for society as a whole? Can molecular diagnostics prevent hospitalization and amputation in a significant number of patients with wound infections? Further, if mNGS detects antibiotic resistance or virulence genes, are these genes expressed, and is it helpful to use this information to select an optimal antibiotic regimen? Currently, these questions remain unanswered.

### Temporal patterns and system dynamics in DFU

In addition to providing results that validate culture, mNGS approaches also have the potential to provide unbiased information on the abundance of organisms in the entire community. This may result in recognizing that knowing which combinations of organisms are present and their relative abundance in the community are associated with improved patient outcomes and the clinical or environmental factors that drive these differences. These complex interactions between microbes, the human host, and the skin environment are key to holistically understanding wound ecology. Yet, capturing the richness of these community dynamics requires temporal sampling to capture the full richness of community interactions. It will also be important to determine if, and how often, wounds should be sampled to determine the microbiome present. The frequency of sampling should probably match the rate of change of the system in order to reveal characteristic fluctuations in microbial communities based on factors that are clinically or environmentally cogent.

To date, most studies of patients with a DFU are limited by both cohort size and sample collection methods and frequency, thus preventing the detection of dynamic dependencies in community composition. In particular, small cohort studies lack statistical robustness and cannot be used worldwide by adjusting for geographic differences [41, 74]. Thus, developing methods to measure and monitor microbial communities in DFU over time to detect their association with patterns of healing may require careful data pooling and machine learning techniques. These techniques could, however, be useful if detecting certain patterns provides clinically relevant early warning signs in wound ecology. These computational methods could provide a deeper understanding of the microbial dynamics in DFU; this, combined with properly applied predictive analytics, could promote targeted and evidence-based therapeutics and patient care with better outcomes.

Before being recommended for widespread clinical use, molecular diagnostics must be carefully validated in the lab to test the limits of detection of microbes, to ensure low rates of false positive or negative findings, and to monitor for contaminants in reagents or poorly obtained or processed microbial samples [35]. Moreover, most studies lack control subjects to ensure any detected differences in microbial community composition and dysbiotic states in ulcers are accurate. To our knowledge, the study by Gardiner et al. is the only one that employed a control group, and they had only eight control subjects [43]. Similarly, there is currently no standard protocol for processing samples and consistently analyzing them. As such, results from relevant studies are inconsistent, and the differences in microbial diversity and composition they demonstrate may be due to biases rather than true biological differences. For example, factors such as patient demographics, clinical characteristics, sampling and sequencing protocols, and downstream analytical methods vary widely across studies. Most importantly, metagenomic studies rarely report the types of wounds and treatment characteristics. In particular, knowing about the performance of wound debridement, the grade of the wound, and the depth of infection is critical to allow comparisons and meta-analyses of these studies.

Moving molecular diagnostics into the clinic will require first building a standard workflow to translate these advanced techniques into corroborated clinical evidence. Currently, reports from mNGS datasets contain diverse microbes that are not typically observed in culture-based analyses, but we do not know which are clinically relevant. Additionally, virulence factors and antibiotic resistance genes are detected but not quantified based on gene expression levels, making their relevance to clinical decisions also unclear. Lastly, because most physicians are not trained to work with or understand mNGS data, there is a major barrier in interpreting the complex and often contradictory results they provide when it comes to choosing antibiotic therapy. These technologically advanced mNGS methods have given us a broader and more accurate view of the microbiome of wounds. However, it is still difficult to interpret the meaning of these data. Thus, as the most recent guidelines for managing diabetic foot infections suggest [65], we will need further research and refinement before moving into the clinic.

## Conclusions

Diabetic foot ulcers are one of the most common, costly, and severe complications of diabetes that have been regarded as a major public health problem. Accurate identification of causative pathogens is essential for early diagnosis and proper treatment. In this review, we have highlighted the latest developments in defining the microbiology of DFUs unveiled by sequencing technologies with an emphasis on temporal analysis and system dynamics, and discuss both the future promises and current limitations of these approaches. Metagenomics can enable the detection of majority microorganisms compared to traditional culture-based techniques, particularly in terms of not yet cultivable bacteria. Molecular tests conducted during weekly office visits to clean and examine DFUs would allow clinicians to offer personalized treatment and antibiotic therapy. Personalized wound management could reduce healthcare costs, improve quality of life for patients, and recoup lost productivity that is important not only to the patient, but also to healthcare payers and providers. These efforts could also improve antibiotic stewardship and control the rise of “superbugs” vital to global health. Moreover, other recently developed approaches like culturomics may provide a better assessment of minority flora. Future studies using such complementary tools to molecular methods will provide more comprehensive insights into the diabetic foot microbiome.

## Data Availability

Not applicable.

## Abbreviations

SSU rRNA: Small subunit ribosomal gene
mNGS: Metagenomic next-generation sequencing
DGGE: Denatured gradient gel electrophoresis
TGGE: Temperature gradient gel electrophoresis
OTU: Operational taxonomic unit
DFU: Diabetic foot ulcer
DFO: Diabetic foot osteomyelitis
